# Screening and characterization of a novel linear B-cell epitope on orf virus F1L protein

**DOI:** 10.3389/fmicb.2024.1373687

**Published:** 2024-06-21

**Authors:** Zhibang Zhang, Xiaoyan Zhang, Kang Feng, Shufan Ba, Taotao Yang, Jinxiang Gong, Ziyin Yang, Hong Zhang, Zilong Sun, Pengcheng Li

**Affiliations:** ^1^College of Life Sciences and Resource and Environment, Yichun University, Yichun, Jiangxi, China; ^2^Shanxi Key Laboratory of Ecological Animal Science and Environmental Veterinary Medicine, College of Veterinary Medicine, Shanxi Agricultural University, Jinzhong, Shanxi, China; ^3^College of Veterinary Medicine, Shanxi Agricultural University, Jinzhong, Shanxi, China; ^4^Xinyu Mengling Animal Husbandry Development Co., Ltd., Xinyu, Jiangxi, China

**Keywords:** orf virus, F1L protein, monoclonal antibody, linear B-cell epitope, homology analysis

## Abstract

**Background:**

Orf, also known as contagious ecthyma (CE), is an acute, contagious zoonotic disease caused by the orf virus (ORFV). The F1L protein is a major immunodominant protein on the surface of ORFV and can induce the production of neutralizing antibodies.

**Methods:**

The prokaryotic expression system was used to produce the recombinant F1L protein of ORFV, which was subsequently purified and used to immunize mice. Positive hybridoma clones were screened using an indirect enzyme-linked immunosorbent assay (ELISA). The reactivity and specificity of the monoclonal antibody (mAb) were verified through Western blot and indirect immunofluorescence (IFA). The linear antigenic epitope specific to the mAb was identified through Western blot, using truncated F1L proteins expressed in eukaryotic cells. A multiple sequence alignment of the ORFV reference strains was performed to evaluate the degree of conservation of the identified epitope.

**Results:**

After three rounds of subcloning, a mAb named Ba-F1L was produced. Ba-F1L was found to react with both the exogenously expressed F1L protein and the native F1L protein from ORFV-infected cells, as confirmed by Western blot and IFA. The mAb recognized the core epitope ^103^CKSTCPKEM^111^, which is highly conserved among various ORFV strains, as shown by homologous sequence alignment.

**Conclusion:**

The mAb produced in the present study can be used as a diagnostic reagent for detecting ORFV and as a basic tool for exploring the mechanisms of orf pathogenesis. In addition, the identified linear epitope may be valuable for the development of epitope-based vaccines.

## Introduction

Orf, also called sore mouth, contagious pustular dermatitis, or contagious ecthyma, is an acute, highly contagious, and epitheliotropic zoonotic disease caused by orf virus (ORFV) (Santiago et al., 2019). ORFV primarily infects domestic ruminants, such as goats and sheep, as well as wild ruminants like deer and camels. It can also infect mammals such as cats and dogs, and in rare cases, humans (Tack and Reynolds, 2011; Spyrou and Valiakos, 2015; Yu et al., 2023). The typical symptoms of orf in animals are ulcerating lesions on the skin and mucous membranes of the mouth, lips, nose, and udders (Du et al., 2023). The morbidity rate of orf is high, particularly in young animals. Fortunately, the fatality rate is usually low unless there are secondary infections (Demiraslan et al., 2017). However, orf could be fatal in lambs and kids due to oral lesions that may impede suckling, and lead to secondary bacterial, viral, fungal, or parasitic infections (Hosamani et al., 2009).

Orf not only has a considerable economic impact on the farming sector but also poses a threat to public health. It can be transmitted from animals to humans through direct or indirect contact with infected animals or contaminated animal products (Andreani et al., 2019; Alajlan and Alsubeeh, 2020). Cases of human-to-human transmission of orf are rare but do occur (Turk et al., 2014; Rajkomar et al., 2016; Bouscarat and Descamps, 2017). Clinical manifestation of human orf usually begins with small papules, which then progress into severe pustular dermatitis lesions, most commonly on the hands and arms (Khan et al., 2021; Qiu and Yanes, 2021). Although orf in humans is a benign and self-limiting disease, it can become complicated in patients with compromised immune systems (Lederman et al., 2007).

Orf virus, the causative agent of orf, is the prototype member of the genus *Parapoxvirus* of the family Poxviridae (Chen et al., 2017). The genome of ORFV is a double-strand linear DNA of about 138 kb with a high G + C content that up to 64% (Spyrou and Valiakos, 2015). Its genome contains 132 putative open reading frames, which are further classified into the central core area (ORFs009-111) and the terminal areas (ORFs001-008 and ORFs112-132) (Li et al., 2023). The genes in the core region are typically associated with virus replication, packaging, morphogenesis, and release, and are relatively conserved (Zhou et al., 2022). On the other hand, the terminal regions, which consist of two inverted repeats are highly variable and are mainly related to virulence, host range, and immune regulation (Bergqvist et al., 2017).

The ORF059 gene is a highly conserved gene located in the core region of the ORFV genome. It is commonly used as a molecular target for phylogenetic analysis of ORFV (Li et al., 2023). The F1L protein, encoded by ORF059, is a significant immunogenic protein that not only triggers the production of neutralizing antibodies (Scagliarini et al., 2002; Gallina et al., 2004), but also may contribute to the induction of cellular immunity (Wang et al., 2022; Zheng et al., 2024). The F1L protein has been shown to have a significant role in the adsorption and entry of the virus into host cells, owing to its ability to bind with heparin (Scagliarini et al., 2004). Furthermore, F1L is a suitable candidate for designing diagnostic methods and subunit vaccines for orf (Yogisharadhya et al., 2018). However, information on the structure and functions of F1L and its antigenic epitopes needs to be further explored. Currently, research on antigenic epitopes of ORFV F1L protein is limited, and there is a wide potential application for the diagnosis of clinical cases using F1L linear B-cell epitope. In order to bridge the gaps between the two, this study aimed to develop and characterize an anti-F1L monoclonal antibody (mAb) and identify the specific antigenic epitope on the F1L protein. These findings have the potential to aid in the diagnosis of orf infection and the development of subunit vaccines against ORFV.

## Materials and methods

### Cell lines, viruses, and plasmids

The SP2/0 myeloma cell line, HEK293T cell line, and immortalized goat endometrial epithelial cells (iGEEC) were maintained in our laboratory and cultured at 37°C in a humidified incubator with 5% CO_2_. The basal medium for the SP2/0 myeloma cell line is RPMI-1640 (Share-bio, China). The basal medium for HEK293T and iGEEC is Dulbecco’s modified Eagle medium (Invitrogen, USA). The cells in this study were cultured in basal medium supplemented with 10% FBS (Gibco, USA), 100 IU/ml penicillin, and 100 μg/ml streptomycin. The ORFV HCE isolate is an attenuated vaccine strain purchased from Shandong Huahong Biotechnology Company Limited. The prokaryotic expression vector pET-28a, the eukaryotic expression vector pEGFP-C1, and pCMV-HA were kept in our laboratory.

### Determination of the antigenicity, hydrophilicity, and transmembrane regions

The characteristics of the ORFV F1L protein were analyzed using Lasergene v7.0 software (Lasergene, USA). The molecular weight (MW) and amino acid (aa) sequence of the cloned ORF059 (F1L) gene were analyzed by the EditSeq program of the Lasergene software. The secondary structure of the F1L protein was analyzed using the Protean program of the Lasergene software. The analysis focused on two aspects: antigenicity and hydrophilicity, which are important for the exogenous expression of the target proteins in ***Escherichia coli***. The potential transmembrane domains of the F1L protein were predicted using the online program TMHMM.^1^

### Preparation and purification of recombinant F1L protein expressed in prokaryotic cells

The prokaryotic expression system was used to express a truncated form of the F1L protein, designated as tF1L, covering amino acids 1–281. The F1L gene was amplified from a sample of orf collected in the field. The F1L gene has been deposited in GenBank with the accession number OQ686990. The tF1L gene was amplified using a pair of primers (tF1L-F and tF1L-R) (Supplementary Table 1) carrying the restriction enzyme sites *Hin*dIII or *Xho*I. The amplified gene was then digested with the appropriate restriction enzymes and ligated to the plasmid pET-28a between the *Hin*dIII and *Xho*I cleavage sites. The recombinant plasmid pET-28a-tF1L was confirmed by sequencing and subsequently transformed into competent BL21(DE3) cells (Tolobio, China). The single positive colony of pET-28a-tF1L transformed BL21(DE3) was induced to express the recombinant protein His-tF1L. The expression of His-tF1L was confirmed by Coomassie Brilliant Blue staining. The SDS-PAGE coupled gel-cutting method was used to purify His-tF1L from the inclusion bodies of the induced *E. coli* lysates, following the procedure described by Han et al. (2019). Briefly, after separation by SDS-PAGE, the protein samples were stained with 0.25 M KCl for approximately 5 min. The proteins with different MWs appeared as white bands in the polyacrylamide gels. The protein bands corresponding to the position of His-tF1L were excised, and homogenized, and an appropriate amount of PBS was added to the gels. After several cycles of freezing and thawing, a portion of the target proteins migrated from the gels into the PBS. The PBS was then separated by centrifugation and aspirated. The purified His-tF1L was identified by staining with Coomassie Brilliant Blue and Western blot analysis using an anti-His mAb (Biodragon, China).

### Indirect enzyme-linked immunosorbent assay

Enzyme-linked immunosorbent assay (ELISA) plates were coated with the purified His-tF1L protein (1 μg/ml, 100 μl/well) and incubated at 4°C overnight. The plates were then blocked with 5% skim milk in PBS at 37°C for 1 h, washed three times with PBS containing 0.05% Tween-20 (PBST), and incubated with hybridoma culture supernatant at 37°C for 1 h. After three washes with PBST, HRP-labeled goat anti-mouse IgG (Beyotime, China) was added to each well at a dilution of 1:3,000 and incubated at 37°C for 1 h. After washing with PBST, 100 μl/well of TMB substrate (Beyotime, China) was added and the color was allowed to develop for 5 min at 37°C. To stop the reaction, 50 μl of 2 M H_2_SO_4_ was added to each well. Absorbance was measured at 450 nm using Multiskan FC (Thermo, USA).

### Production and characterization of murine mAb against F1L protein

Monoclonal antibodies against the recombinant F1L protein of ORFV were generated according to a previously described procedure (Zhang, 2012). Three female BALB/c mice, aged 7 weeks, were immunized three times with 100 μg of purified His-tF1L via subcutaneous injection at 21-day intervals. The antigen was emulsified with complete Freund’s adjuvant for the first dose and incomplete Freund’s adjuvant for the subsequent booster immunizations (Sigma, USA). The antibody levels of the immunized mice were assessed through indirect ELISA after three immunizations, using purified His-tF1L as the coat antigen. A booster dose of 100 μg His-tF1L protein without adjuvant was administered intraperitoneally to the mouse with the highest antibody level 3 days before cell fusion. The mouse selected for the experiment was euthanized and its splenocytes were fused with SP2/0 myeloma cells according to standard procedures (Galfre and Milstein, 1981). The resulting hybridoma cell supernatants were tested for His-tF1L antibodies by indirect ELISA. The hybridoma cells were subcloned three times by limiting dilution and verified as His-tF1L specific antibody positive by ELISA. The purified hybridoma cells were then injected into the peritoneal cavity of BALB/c mice to generate ascites fluid against the F1L protein. The isotype of the F1L mAb was determined using a Mouse Monoclonal Antibody Ig Class/subclass Identification ELISA Kit (Biodragon, China) and an IsoQuick Strips and Kits for Mouse Monoclonal Isotyping (Sigma-Aldrich, MO, USA) according to the manufacturer’s protocol. The reactivity of the F1L mAb with ORFV were evaluated using Western blot analysis and immunofluorescence assay (IFA).

### Expression of full or a series of truncated F1L proteins in eukaryotic cells

To determine the minimal epitope recognized by the F1L mAb, the amino acids at both ends of the tF1L protein were gradually deleted until the core epitope was identified. A series of truncated F1L proteins that were used in the study are shown in Figures 4A, 5A. The genes encoding the truncated forms of the F1L protein were inserted into the pEGFP-C1 plasmid via *Hin*dIII and *Kpn*I restriction sites. The HEK293T cells were transfected with the recombinant plasmids pEGFP-C1-tF1L or its derivatives to express the F1L protein and its various truncations. Simply, when the cell culture reached 70%–80% confluence, 2.5 μg of each recombinant plasmid was transfected into HEK293T cells in a six-well plate. Cells were harvested at 48 h post-transfection, and the expression of each GFP-fused protein was confirmed through Western blot analysis using both GFP mAb and F1L mAb.

For pCMV-HA-F1L construction, the intact ORF059 gene was amplified using the primers HA-F1L-F and HA-F1L-R (Supplementary Table 1), containing *Eco*RI and *Xho*I restriction sites, respectively. The resultant amplicon was inserted into the eukaryotic expression plasmid pCMV-HA via *Eco*RI/*Xho*I restriction sites to generate the recombinant plasmid pCMV-HA-F1L. For *in vitro* expression of HA-F1L, HEK293T cells were transfected with recombinant plasmid pCMV-HA-F1L as described above.

### Indirect immunofluorescence assay

The expression of HA-F1L protein in HEK293T cells was assessed through IFA 36 h after transfection. The native F1L protein was obtained by infecting iGEEC with ORFV HCE strain at an MOI of 0.5. Briefly, iGEEC seeded in glass-bottomed culture dishes at approximately 90% confluence were inoculated with ORFV. When there were obvious cytopathic effects after infection, cells were fixed with 4% paraformaldehyde (Biosharp, China) in PBS for 30 min at room temperature, and rinsed three times with PBS for 5 min each time. The fixed cells were then permeabilized with 0.1% Triton X-100 (Macklin, China) in PBS/2% BSA (Saiguo, China) for 10 min. The liquid was aspirated and the cells were blocked with PBS/2% BSA for 30 min at 37°C. After washing with PBS, the cells were incubated with mAb Ba-F1L (1:500 dilution) for 1 h at 37°C. After washing, cells were incubated with donkey anti-mouse IgG antibody (1:1,000) conjugated to FITC (Share-bio, China) for 1 h at 37°C. Cells were washed thrice with PBS and stained with DAPI (Sigma, USA) for 10 min at room temperature before final washing with PBS. Images were captured by fluorescence microscopy (Nikon, Japan).

### Western blot analysis

Western blot was used to analyze the reactivity of mAb Ba-F1L with viral native F1L and GFP-fused F1L protein for the fine determination of the core epitope. Culture lysates containing native F1L or GFP-fused F1L polypeptides were subjected to 10% SDS-PAGE followed by transfer to polyvinylidene fluoride (PVDF) membrane. After blocking with 5% skim milk in PBS at 37°C for 1 h, PVDF membranes were incubated with mAb Ba-F1L (1:500 dilution) or GFP (1:1,000 dilution, Share-bio, China) at 4°C overnight. After three washes with PBS containing 0.05% Tween-20 (PBST), the membranes were incubated with HRP-conjugated goat anti-mouse IgG (1:3,000 dilution, Share-bio, China) at room temperature for 1 h. The membranes were washed with PBST and developed using an ECL imaging system (Amersham Imager 600, General Electric, USA).

### Sequence analysis

To analyze whether the epitope identified in the present study is conservative among different ORFV strains, the epitope sequences and their flanking sequences of the F1L proteins were aligned among 17 ORFV reference strains using BioEdit software V7.2.5.

## Results

### Amino acid characteristics prediction results

**FIGURE 1:**
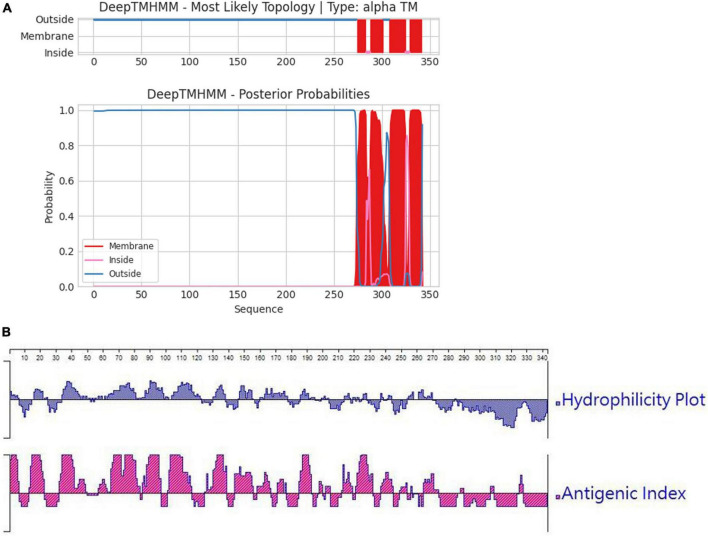
Predicted properties of the F1L protein. **(A)** The transmembrane regions of the F1L protein were forecasted using the online program TMHMM, with the red lines denoting the identified transmembrane domains. **(B)** Hydrophilicity and antigenicity analysis of the full-length F1L protein using the Protean program of the Lasergene software.

In order to ascertain the antigenicity, hydrophilicity, MW, and transmembrane region of the F1L protein, we initially conducted a targeted analysis using bioinformatics software. The results are depicted in Figures 1A, B. It was revealed that the net MW of tF1L is approximately 31 kDa. In comparison to the full-length F1L protein, the N-terminal 281 amino acids (tF1L) exhibit higher immunogenicity and hydrophilicity. The F1L protein has multiple transmembrane regions at its C-terminus. Consequently, we chose to express the N-terminal 281 amino acids (tF1L) of the F1L protein instead of the full form F1L.

### Expression and purification of recombinant His_6_-tF1L

**FIGURE 2:**
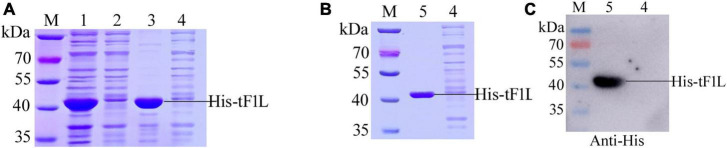
Expression, purification, and identification of the recombinant His-tF1L protein. Bacterial lysates from *E. coli* transformed with recombinant plasmid pET-28a-tF1L or empty plasmid pET-28a were subjected to SDS-PAGE analysis **(A)**. Analysis of the purified His-tF1L protein by Coomassie blue staining **(B)** and Western blot **(C)**. Lane M, protein molecular weight maker; lanes 1–3, the whole cell lysates, the supernatant and the precipitation of *E. coli* cells transformed with the recombinant plasmid pET-28a-tF1L; lane 4, the whole cell lysates of *E. coli* cells transformed with the empty plasmid pET-28a; lane 5, the purified His-tF1L protein.

The prokaryotic expression vector pET-28a was used as the vehicle to express the F1L protein. In the present study, tF1L was expressed as a fusion protein in the form of hexahistidine-tF1L-hexahistidine. The MW of the recombinant protein His-tF1L, as estimated by the software lasergene, is approximately 37 kDa. As shown in Figure 2A, the His-tF1L protein was successfully expressed and almost all the target proteins were present in an insoluble fraction. However, the actual MW of the prokaryotic form of His-tF1L is a slightly larger than the deduced MW. We purified the insoluble His-tF1L by a method called “SDS-PAGE and gel-cutting” as described previously (Han et al., 2019). The purity of purified His-tF1L was relatively high as shown by SDS-PAGE (Figure 2B). Western blot analysis showed that the purified His-tF1L could be recognized by the anti-His mAb (Figure 2C).

### Production and characterization of F1L protein-specific mAb

**FIGURE 3:**
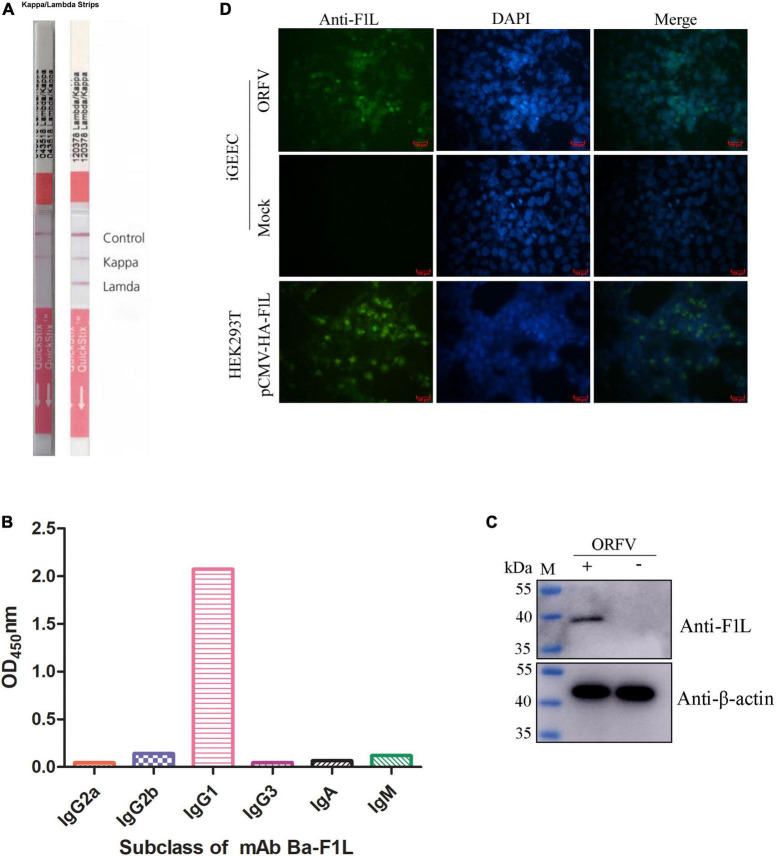
Characterization of the F1L mAb. **(A)** Isotyping the mAb light chain. **(B)** Isotyping the mAb heavy chain. **(C)** Reactivity of the mAb by Western blot. The iGEEC were infected with ORFV HCE isolate or mock infection. Cell lysates were subjected to immunoblotting by incubation with anti-F1L mAb and β-actin mAb. Lane M, protein maker. **(D)** Reactivity of the mAb by IFA. The iGEEC were infected with ORFV or mock infection. When CPE appeared in virus-infected cells, the anti-F1L mAb was used as the primary antibody to perform IFA. HEK293T cells were transfected with the recombinant plasmid pCMV-HA-F1L. The mAb Ba-F1L was used to detect the expression of HA-F1L protein at 36 h post-transfection.

The purified His-tF1L protein was used both as an immunogen and as a screening antigen for the development of hybridomas. Positive clones were screened from hybridoma supernatants using purified His-tF1L protein as the coating antigen in an indirect ELISA. After three rounds of screening and subcloning, a hybridoma cell line that secretes F1L protein-specific antibodies was selected and designated Ba-F1L. Ascites fluid was produced by infecting mice with the Ba-F1L hybridoma cell line. The isotype of the mAb Ba-F1L light chain was determined using an IsoQuick Strips and Kits for Mouse Monoclonal Isotyping. The right strip in Figure 3A has been extracted from the instructions accompanying the Strips and Kits, which provide guidance on how to judge the results. As shown in Figure 3A, the left strip developed a test line and a kappa line, which indicated that the light chain of mAb Ba-F1L was κ. The heavy chain subclasses were identified by another commercial kit. The results clearly indicated that the mAb Ba-F1L heavy chain was IgG1, as demonstrated by testing at a dilution of 1:100,000 of the ascites (Figure 3B).

To assess the reactivity of the mAb Ba-F1L, we used Western blot and IFA to test its ability to recognize the F1L protein. As shown in Figure 3C, a specific band at the position of the expected MW of F1L (approximate 39 kDa) was present in ORFV-infected iGEEC but not in mock-infected cells. Green fluorescence signals were observed in both ORFV-infected iGEEC and HEK293T cells transfected with the eukaryotic plasmid pCMV-HA-F1L when using the mAb Ba-F1L as the primary antibody in IFA. However, no fluorescence signals were present in mock-infected iGEEC (Figure 3D). The fluorescence signals of HEK293T cells transfected with recombinant plasmids are stronger than those of iGEEC infected with ORFV. This may be due to the higher expression level of exogenous F1L in HEK293T compared to the viral native F1L protein in iGEEC.

### Preliminary determination of the antigenic epitope domain

**FIGURE 4:**
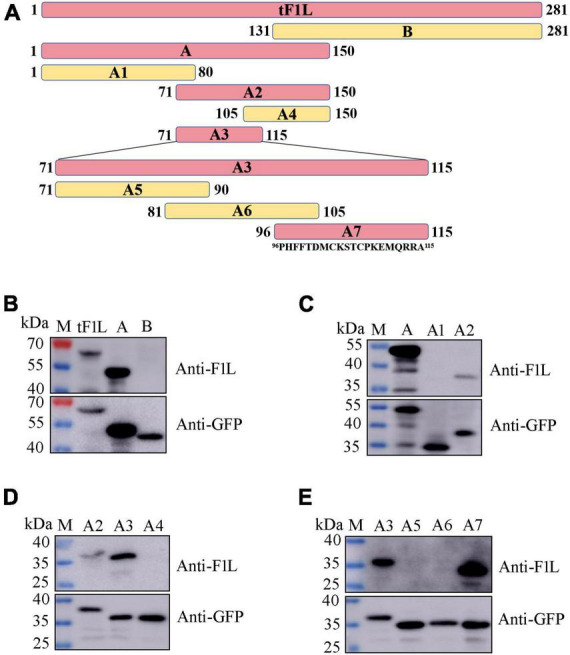
Identification of the epitope-containing domain by Western blot. **(A)** Schematic representation of the F1L protein fragments used for B-cell epitope mapping. The length of each fragment is indicated by numbers at both ends. The amino acid residues of fragment A7 are shown. **(B–E)** Preliminary identification of the B-cell epitope in the F1L protein by Western blot. The samples in each lane are indicated. Lane M, protein maker.

The basic strategy for identifying the epitope-containing domain in F1L involves progressively splitting and truncating the positive proteins/polypeptides that react with mAb Ba-F1L. In this section, a series of four successive rounds of overlapping GFP-tagged proteins or polypeptides were constructed and expressed in HEK293T cells through transfection of the corresponding recombinant plasmids (Figure 4A). The tF1L protein is 281 aa in length. For the first round, the tF1L protein was divided into two parts, fragment A (150 aa) and fragment B (151 aa), with an overlap portion of 20 aa. The anti-GFP mAb recognized the tF1L protein, fragments A, and B, all of which bear a GFP tag (Figure 4B). However, only the tF1L protein and fragment A exhibited reactivity with the mAb, thereby signifying that the specific epitope recognized by mAb Ba-F1L was situated within fragment A (Figure 4B). Subsequently, over the course of the three rounds of epitope mapping, fragments A2, A3, and A7 were successively identified as the epitope-containing domains (Figures 4C–E). Consequently, it can be concluded that the epitope-containing region of the F1L protein is located between amino acids 96 and 115.

### Precise mapping of mAb Ba-F1L-specific epitope

**FIGURE 5:**
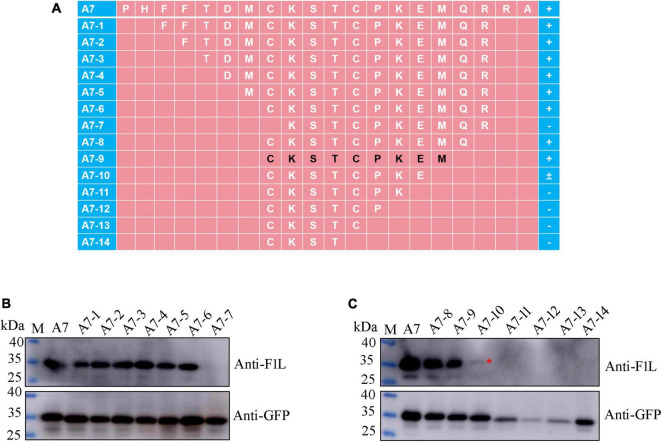
Precise mapping of the mAb-specific minimal epitope by Western blot. **(A)** Schematic representation of the polypeptides of the F1L protein used to map B-cell epitope. The amino acid residues corresponding to each polypeptide are shown, and the core epitope finally identified in this study is marked in black. The names of the polypeptides are shown to the left of panel **(A)**. The positive reaction, negative reaction, and weak reaction between the polypeptides and the mAb is indicated by the plus (+), minus (–), or plus-minus (±) signs to the right of panel **(A)**. **(B,C)** Identification of the minimal B-cell epitope recognized by the F1L mAb. The samples in each lane are indicated. Lane M, protein maker. The red asterisk in panel **(C)** points to the weak Western blot signal.

For fine mapping of the core epitope, a stepwise deletion of amino acids was carried out from either the N-terminal or C-terminal of fragment A7 (96–115 aa), resulting in a total of 14 polypeptides derived from A7 (Figure 5A). These polypeptides were expressed as GFP-fused proteins through the transient transfection of the corresponding plasmids into HEK293T cells. Subsequently, Western blot analysis was conducted on lysates from cells transfected with each recombinant plasmid to assess their reactivity with either the GFP mAb, F1L mAb, or both. As depicted in Figures 5B, C, all polypeptides exhibited reactivity with the GFP mAb, indicating the successful expression of each recombinant protein. Notably, the polypeptide A7-7 failed to be recognized by the F1L mAb, implying the essential nature of the cysteine at position 103 in F1L for the mAb-specific epitope (Figure 5B). Polypeptides A7-8 to A7-14 share the same amino acid ^103^C in their N-terminus, differing solely in their C-terminus due to the progressive deletion of one amino acid at a time (Figure 5A). In Figure 5C, it is evident that polypeptides A7-8 and A7-9 exhibited a strong reaction with the F1L mAb, whereas A7-10 only yielded a weak signal. The remaining polypeptides, A7-11 to A7-14, showed no reactivity with the F1L mAb (Figure 5C). The polypeptide ^103^CKSTCPKE^110^ displayed weak reactivity with the mAb Ba-F1L. However, the addition of the amino acid M^111^ to the polypeptide ^103^CKSTCPKE^110^ resulted in a strong reactivity with the F1L mAb, as observed in sample A7-9 in Figure 5C. These findings indicate that the core epitope recognized by the mAb Ba-F1L is ^103^CKSTCPKEM^111^ within the F1L protein.

### Homology analysis of the F1L epitope in ORFV strains

**FIGURE 6:**
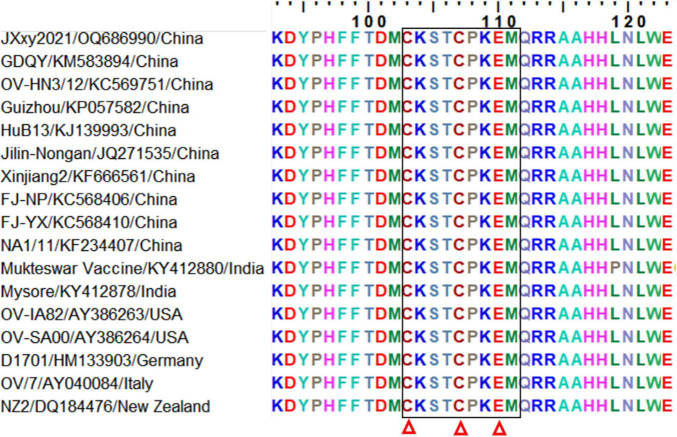
Amino acid sequence alignment of the defined epitope and its flanking regions among different representative ORFV strains. A dozen reference sequences of the F1L protein were aligned. The information for each sequence, including names, GenBank accession numbers, and countries, is shown. The black box indicates the epitope identified in this study. The three red triangles at the bottom of the figure indicate the conserved cysteines and glutamic acid in ORFV F1L protein and its homolog in the poxvirus family.

Sequence alignment was performed to determine the conservation level of the epitope recognized by mAb Ba-F1L among 17 representative ORFV strains from China and other countries (Table 1). The results showed that the epitope ^103^CKSTCPKEM^111^ exhibits complete conservation across all ORFV reference strains, both domestic and abroad (Figure 6). The polypeptide ^103^CKSTCPKEM^111^ represents a highly conserved epitope on the F1L protein of ORFV, indicating the mAb Ba-F1L has the potential to react with any ORFV strain globally.

**TABLE 1:** The F1L genes of ORFV reference strains used in this study.

Strains	GenBank no.	Host	Country	Year
Jxxy2021	OQ686990	Goat	China	2021
GDQY	KM583894	Goat	China	2014
OV-HN3/12	KC569751	Sheep	China	2012
Guizhou	KP057582	Goat	China	2010
HuB13	KJ139993	Goat	China	2013
Jilin-Nongan	JQ271535	Sheep	China	2011
Xinjiang2	KF666561	Goat	China	2013
FJ-NP	KC568406	Goat	China	2011
FJ-YX	KC568410	Goat	China	2012
NA1/11	KF234407	Sheep	China	2011
Mukteswar vaccine	KY412880	Goat	India	2005
Mysore	KY412878	Sheep	India	2010
OV-IA82	AY386263	Sheep	USA	1982
OV-SA00	AY386264	Goat	USA	2003
D1701	HM133903	Sheep	Germany	2010
OV/7	AY040084	Sheep	Italy	2001
NZ2	DQ184476	Sheep	New Zealand	2005

## Discussion

China has the largest population of domestic small ruminants in the world, with an estimated population of over 300 million sheep and goats (Mao et al., 2022). With the rapid development of the sheep and goat-raising industry in recent years, small ruminant-associated diseases have emerged and reemerged in China. These include peste des petits ruminants, caprine parainfluenza type 3, border disease, enzootic nasal tumor, caprine herpesvirus 1 infection, enterovirus infection, and orf (Zhao et al., 2010; Mao et al., 2022). Orf is a zoonotic disease that is distributed globally and has caused significant financial losses in the breeding industry (Li et al., 2023). Additionally, it may have an impact on human health, especially for individuals who come into contact with ORFV-infected animals or their products, such as veterinarians and farmers (Du et al., 2023).

The expeditious and accurate diagnosis of orf is imperative for effective disease control. Initial diagnosis relies heavily on clinical manifestations and epidemiological history (Pang and Long, 2023). However, the accuracy of the initial diagnosis is predominantly contingent upon individual professional expertise, which may not consistently yield reliable results. The differentiation of orf from similar diseases such as foot and mouth disease, peste des petits ruminants, bluetongue disease, and goat pox or sheep pox can be challenging due to clinical similarities and the potential for co-infections (Gou et al., 2022; Pan et al., 2023). Molecular methods such as standard PCR, real-time PCR, virus isolation, ELISA, and electron microscopy are commonly used in the laboratory to confirm the preliminary diagnosis (Pang and Long, 2023). Nevertheless, the implementation of these molecular methods is often hindered on sheep or goat farms in China due to the shortage of skilled personnel to conduct the tests. Consequently, there is an urgent need for a straightforward and convenient diagnostic approach, such as an immunocolloidal gold test strip. However, the immunocolloidal gold test strips for the detection of ORFV are not commercially available. The development of immunocolloidal gold test strips necessitates monoclonal antibodies with high specificity and affinity, which are also valuable for various molecular immunology techniques utilized in fundamental research. Hence, we embarked on the development of the mAb against ORFV.

F1L protein is the immunodominant envelope antigen of ORFV and participates in viral adsorption and entry of target cells (Czerny et al., 1997; Scagliarini et al., 2004). Several research groups have demonstrated the potential utility of the F1L protein as a candidate for subunit vaccines against various ORFV strains (Zhao et al., 2011; Wang et al., 2022, 2023). Considering the important roles played by F1L, it is essential to develop F1L mAbs and identify its antigenic epitopes. Czerny et al. (1997) generated several mAbs against ORFV, and one of the mAbs (named 4D9) exhibited high affinity for the 39 kDa protein in purified ORFV particles. The mAb 4D9 had neutralizing capacity and was able to recognize the F1L protein in Western blots (Scagliarini et al., 2002). Another group developed a panel of 27 mouse mAbs against ORFV, of which 8 mAbs could react with native F1L protein (Housawi et al., 1998). The above-mentioned F1L mAbs were produced using the purified ORFV particles as immunogen. In another report, 10 mAbs were generated by immunizing mice with bacterially expressed F1L protein. Two mAbs (A3 and G3) with the greatest reactivity among these 10 mAbs were shown to react with viral native F1L protein (Li et al., 2012). The mAb developed in this study was also able to recognize naturally and artificially expressed F1L protein, both in IFA and Western blot analysis.

The bacterium *E. coli* is the most widely used vehicle for the overexpression of exogenous proteins. The ORF059 gene cloned in this study (GenBank No. OQ686990) is 1,029 bp long and the deduced amino acids of the F1L protein are 342. At the beginning of the study, we cloned the entire ORF059 gene into a bacterial expression vector pET-28a. However, the expression level of the full-length F1L protein was very low, even when different expression conditions were tested, such as incubation temperatures, induction times, and IPTG concentrations. This may be due to the properties of the F1L protein. The F1L protein has multiple transmembrane domains at its C-terminal as predicted by the online program (TMHMM) for transmembrane prediction. It is widely recognized that exogenous proteins containing transmembrane domains are typically expressed at low levels or not at all in *E. coli* (Wagner et al., 2007). We decided to express the N-terminal 281 amino acids of the F1L protein (tF1L), from which almost all the transmembrane regions of the F1L protein have been eliminated. At the same time, the tF1L protein retained good antigenicity and hydrophilicity, making it suitable for use as an immunogen. The tF1L protein was successfully expressed and its expression level was relatively high. For the same reason, part of the F1L protein was expressed in *E. coli* and an indirect ELISA was established based on the rF1L protein, which lacks the transmembrane regions (289–334 aa) (Yogisharadhya et al., 2018). In contrast, the intact F1L protein was highly expressed in another report using the bacterial expression plasmid pET28a-ORF059 (Li et al., 2012). The inconsistency observed in the expression patterns of the full-length F1L protein in *E. coli* across various research groups remains ambiguous and necessitates thorough investigation.

The ORFV F1L protein is a homolog of the vaccinia HL3 protein, the Capripoxvirus P32 protein, and the lumpy skin disease virus P32 protein because of their structural similarities (Housawi et al., 1998; Heine et al., 1999). A pair of cysteine residues at 103 and 107 and a glutamic acid at 110 of the identified epitope ^103^CKSTCPKEM^111^ are highly conserved and identical in the ORFV F1L protein as well as in the F1L homologs of animal poxviruses (Lin et al., 2000). In other words, other viruses in the Poxviridae family have at least five or six different amino acids in their counterparts of the ORFV F1L protein epitope ^103^CKSTCPKEM^111^. As the epitope sequence identified in this study varies widely among different poxviruses, it is plausible that the mAb Ba-F1L may not interact with sheep poxvirus, goat poxvirus, and other viruses in the Poxviridae family. Consequently, the monoclonal antibody Ba-F1L holds potential utility in devising a methodology to differentiate ORFV from other poxviruses.

## Conclusion

A novel mAb directed against ORFV was successfully generated, with the mAb-specific linear epitope identified at residues ^103^CKSTCPKEM^111^ within the F1L protein. This report presents the first identification of a linear antigenic epitope on the ORFV F1L protein. Owning to the high conservation of the identified epitope among ORFV isolates, the mAb exhibits the potential to serve as a universal diagnostic tool for all ORF viruses. Furthermore, it holds promise for contributing to elucidating the structural and functional aspects of the F1L protein through future investigations. Moreover, the epitope’s characteristics could contribute to the development of epitope-based vaccines.

## Data availability statement

The datasets presented in this study can be found in online repositories. The names of the repository/repositories and accession number(s) can be found in the article/Supplementary material.

## Ethics statement

The animal study was approved by the Animal Care and Ethics Committee of Yichun University. The study was conducted in accordance with the local legislation and institutional requirements.

## Author contributions

ZZ: Investigation, Writing – original draft, Conceptualization, Funding acquisition. XZ: Investigation, Funding acquisition, Writing – review & editing. KF: Investigation, Writing – review & editing. SB: Investigation, Writing – review & editing. TY: Formal analysis, Funding acquisition, Software, Writing – review & editing. JG: Validation, Writing – review & editing. ZY: Data curation, Writing – review & editing. HZ: Data curation, Resources, Writing – review & editing. ZS: Project administration, Supervision, Writing – review & editing. PL: Conceptualization, Funding acquisition, Project administration, Writing – original draft.

## Footnotes

1
https://dtu.biolib.com/DeepTMHMM

## References

[B1] Alajlan A. M. Alsubeeh N. A. (2020). Orf (ecthyma contagiosum) Transmitted from a camel to a human: A case report. *Am. J. Case Rep.* 21:e927579. 10.12659/ajcr.927579 33353926 PMC7768592

[B2] Andreani J. Fongue J. Bou Khalil J. Y. David L. Mougari S. Le Bideau M. (2019). Human infection with orf virus and description of its whole genome, France, 2017. *Emerg. Infect. Dis.* 25 2197–2204. 10.3201/eid2512.181513 31742503 PMC6874271

[B3] Bergqvist C. Kurban M. Abbas O. (2017). Orf virus infection. *Rev. Med. Virol.* 27:e1932. 10.1002/rmv.1932 28480985

[B4] Bouscarat F. Descamps V. (2017). Wife to husband transmission of ecthyma contagiosum (Orf). *IDCases* 9 28–29. 10.1016/j.idcr.2017.05.007 28560176 PMC5447513

[B5] Chen H. Li W. Kuang Z. Chen D. Liao X. Li M. (2017). The whole genomic analysis of orf virus strain HN3/12 isolated from Henan province, central China. *BMC Vet. Res.* 13:260. 10.1186/s12917-017-1178-1 28821255 PMC5562994

[B6] Czerny C. P. Waldmann R. Scheubeck T. (1997). Identification of three distinct antigenic sites in parapoxviruses. *Arch. Virol.* 142 807–821. 10.1007/s007050050120 9170506

[B7] Demiraslan H. Dinc G. Doganay M. (2017). An overwiev of ORF virus infection in humans and animals. *Recent Patents Anti Infect. Drug Discov.* 12 21–30. 10.2174/1574891x12666170602080301 28571550

[B8] Du G. Wu J. Zhang C. Cao X. Li L. He J. (2023). The whole genomic analysis of the Orf virus strains ORFV-SC and ORFV-SC1 from the Sichuan province and their weak pathological response in rabbits. *Funct. Integr. Genom.* 23:163. 10.1007/s10142-023-01079-z 37188892 PMC10185592

[B9] Galfre G. Milstein C. (1981). Preparation of monoclonal antibodies: Strategies and procedures. *Methods Enzymol.* 73 3–46. 10.1016/0076-6879(81)73054-4 7300683

[B10] Gallina L. Ciulli S. Prosperi S. (2004). Cloning and expression of the Orf virus F1L gene possible use as a subunit vaccine. *Vet. Res. Commun.* 1 291–293. 10.1023/b:verc.0000045429.78312.f6 15372980

[B11] Gou Q. Du J. Zhu Z. Chen X. Zhao H. Zhao Y. (2022). Establishment and preliminary application of multiplex real-time PCR for detecting bluetongue virus, peste des petits ruminants virus, orf virus and capripoxvirus. *Chin. Vet. Sci.* 52 1510–1517. 10.16656/j.issn.1673-4696.2022.0175

[B12] Han Y. Zhang J. Shi H. Zhou L. Chen J. Zhang X. (2019). Epitope mapping and cellular localization of swine acute diarrhea syndrome coronavirus nucleocapsid protein using a novel monoclonal antibody. *Virus Res.* 273:197752. 10.1016/j.virusres.2019.197752 31518629 PMC7114574

[B13] Heine H. G. Stevens M. P. Foord A. J. Boyle D. B. (1999). A capripoxvirus detection PCR and antibody ELISA based on the major antigen P32, the homolog of the vaccinia virus H3L gene. *J. Immunol. Methods* 227 187–196. 10.1016/s0022-1759(99)00072-1 10485266

[B14] Hosamani M. Scagliarini A. Bhanuprakash V. Mcinnes C. J. Singh R. K. (2009). Orf: An update on current research and future perspectives. *Expert Rev. Anti Infect. Ther.* 7 879–893. 10.1586/eri.09.64 19735227

[B15] Housawi F. M. Roberts G. M. Gilray J. A. Pow I. Reid H. W. Nettleton P. F. (1998). The reactivity of monoclonal antibodies against orf virus with other parapoxviruses and the identification of a 39 kDa immunodominant protein. *Arch. Virol.* 143 2289–2303. 10.1007/s007050050461 9930187

[B16] Khan Y. Currie J. Miller C. Lawrie D. (2021). Orf virus infection of the hand in a Scottish sheep farmer. A case report to increase awareness to avoid misdiagnosis. *Case Rep. Plast. Surg. Hand Surg.* 9 26–29. 10.1080/23320885.2021.2016057 34926720 PMC8676690

[B17] Lederman E. R. Green G. M. Degroot H. E. Dahl P. Goldman E. Greer P. W. (2007). Progressive orf virus infection in a patient with lymphoma: Successful treatment using imiquimod. *Clin. Infect. Dis.* 44 e100–e103. 10.1086/517509 17479930

[B18] Li H. Ning Z. Hao W. Zhang S. Liao X. Li M. (2012). Identification and characterization of monoclonal antibodies against the ORFV059 protein encoded by Orf virus. *Virus Genes* 44 429–440. 10.1007/s11262-011-0710-9 22237464

[B19] Li S. Jing T. Zhu F. Chen Y. Yao X. Tang X. (2023). Genetic analysis of orf virus (ORFV) strains isolated from goats in China: Insights into epidemiological characteristics and evolutionary patterns. *Virus Res.* 334:199160. 10.1016/j.virusres.2023.199160 37402415 PMC10410590

[B20] Lin C. L. Chung C. S. Heine H. G. Chang W. (2000). Vaccinia virus envelope H3L protein binds to cell surface heparan sulfate and is important for intracellular mature virion morphogenesis and virus infection in vitro and in vivo. *J. Virol.* 74 3353–3365. 10.1128/jvi.74.7.3353-3365.2000 10708453 PMC111837

[B21] Mao L. Li W. Hao F. Yang L. Li J. Sun M. (2022). Research progress on emerging viral pathogens of small ruminants in china during the last decade. *Viruses* 14:1288. 10.3390/v14061288 35746759 PMC9228844

[B22] Pan Y. Yang L. Li T. Mo B. Li G. (2023). Establishment and application of multiplex PCR detection methods for foot and mouth disease virus, orf virus and goat pox virus. *J. Anhui Agric. Sci.* 51 112–115. 10.3969/j.issn.0517-6611.2023.21.026

[B23] Pang F. Long Q. (2023). Recent advances in diagnostic approaches for orf virus. *Appl. Microbiol. Biotechnol.* 107 1515–1523. 10.1007/s00253-023-12412-8 36723701

[B24] Qiu C. S. Yanes A. F. (2021). Orf. *N. Engl. J. Med.* 384:1552. 10.1056/NEJMicm2029641 33882630

[B25] Rajkomar V. Hannah M. Coulson I. H. Owen C. M. (2016). A case of human to human transmission of orf between mother and child. *Clin. Exp. Dermatol.* 41 60–63. 10.1111/ced.12697 26299382

[B26] Santiago L. Oliveira D. Cardoso J. C. Figueired A. (2019). Human orf: An under-recognized entity. *Acta Dermatovenerol. Croat.* 27 280–281. 31969245

[B27] Scagliarini A. Ciulli S. Battilani M. Jacoboni I. Montesi F. Casadio R. (2002). Characterisation of immunodominant protein encoded by the F1L gene of orf virus strains isolated in Italy. *Arch. Virol.* 147 1989–1995. 10.1007/s00705-002-0850-2 12376759

[B28] Scagliarini A. Gallina L. Dal Pozzo F. Battilani M. Ciulli S. Prosperi S. (2004). Heparin binding activity of orf virus F1L protein. *Virus Res.* 105 107–112. 10.1016/j.virusres.2004.04.018 15351483

[B29] Spyrou V. Valiakos G. (2015). Orf virus infection in sheep or goats. *Vet. Microbiol.* 181 178–182. 10.1016/j.vetmic.2015.08.010 26315771

[B30] Tack D. M. Reynolds M. G. (2011). Zoonotic poxviruses associated with companion animals. *Animals* 1 377–395. 10.3390/ani1040377 26486622 PMC4513476

[B31] Turk B. G. Senturk B. Dereli T. Yaman B. (2014). A rare human-to-human transmission of orf. *Int. J. Dermatol.* 53 e63–e65. 10.1111/j.1365-4632.2012.05669.x 23557377

[B32] Wagner S. Baars L. Ytterberg A. J. Klussmeier A. Wagner C. S. Nord O. (2007). Consequences of membrane protein overexpression in *Escherichia coli*. *Mol. Cell. Proteomics* 6 1527–1550. 10.1074/mcp.M600431-MCP200 17446557

[B33] Wang Y. Sun S. Zhao K. Du L. Wang X. He W. (2023). Orf virus DNA prime-protein boost strategy is superior to adenovirus-based vaccination in mice and sheep. *Front. Immunol.* 14:1077938. 10.3389/fimmu.2023.1077938 37026014 PMC10070790

[B34] Wang Y. Zhao K. Song D. Du L. Wang X. Gao F. (2022). Evaluation of the immune response afforded by combined immunization with orf virus DNA and subunit vaccine in mice. *Vaccines* 10:1499. 10.3390/vaccines10091499 36146577 PMC9504141

[B35] Yogisharadhya R. Kumar A. Bhanuprakash V. Shivachandra S. B. (2018). Evaluation of a recombinant major envelope protein (F1L) based indirect- ELISA for sero-diagnosis of orf in sheep and goats. *J. Virol. Methods.* 261 112–120. 10.1016/j.jviromet.2018.08.015 30149033

[B36] Yu D. Yao K. Deng D. Liu Y. Wu R. Li Y. (2023). A transmission chain from sheep to sheep and human of zoonotic orf virus during the mpox epidemic. *Emerg. Microbes Infect.* 12:2233636. 10.1080/22221751.2023.2233636 37427540 PMC10360991

[B37] Zhang C. (2012). Hybridoma technology for the generation of monoclonal antibodies. *Methods Mol. Biol.* 901 117–135. 10.1007/978-1-61779-931-0_7 22723097

[B38] Zhao K. He W. Gao W. Lu H. Han T. Li J. (2011). Orf virus DNA vaccines expressing ORFV 011 and ORFV 059 chimeric protein enhances immunogenicity. *Virol. J.* 8:562. 10.1186/1743-422X-8-562 22204310 PMC3269396

[B39] Zhao K. Song D. He W. Lu H. Zhang B. Li C. (2010). Identification and phylogenetic analysis of an orf virus isolated from an outbreak in sheep in the Jilin province of China. *Vet. Microbiol.* 142 408–415. 10.1016/j.vetmic.2009.10.006 19948384

[B40] Zheng W. Zhang Y. Gu Q. Liang Q. Long Y. Wu Q. (2024). Development of an indirect ELISA against orf virus using two recombinant antigens, partial B2L and F1L. *J. Virol. Methods* 326:114891. 10.1016/j.jviromet.2024.114891 38336349

[B41] Zhou Y. Guan J. Lv L. Cui H. Xu M. Wang S. (2022). Complete genomic sequences and comparative analysis of two orf virus isolates from Guizhou province and Jilin province, China. *Virus Genes* 58 403–413. 10.1007/s11262-022-01918-4 35780442

