# Supplementary material for: Screening and characterization of a novel linear B-cell epitope on orf virus F1L protein
Source: Front Microbiol. 2024 Jun 21;15:1373687. doi: 10.3389/fmicb.2024.1373687 (PMC11224485; doi:10.3389/fmicb.2024.1373687)
Supplement: Supplementary file 1 [file Table_1.DOCX]

**Table S1. Primers used in this study**

| Primers | Sequeces (5’-3’) | Position (aa)^#^ |
| --- | --- | --- |
| F1L-F | ATGGATCCACCCGAAATCAC | 1-342 |
| F1L-R | TCACACGATGGCCGTGACC |  |
| HA-F1L-F | accgGAATTCggATGGATCCACCCGAAATCAC | 1-342 |
| HA-F1L-R | accgCTCGAGTCACACGATGGCCGTGACC |  |
| tF1L-F | atatAAGCTTgcATGGATCCACCCGAAATC | 1-281 |
| tF1L-R | atatCTCGAGCCACGTGGCGAGCAGCGAC |  |
| A-F | acccAAGCTTcgATGGATCCACCCGAAATCAC | 1-150 |
| A-R | acggGGTACCGCGGAAGGCCATGTCGTTGTC |  |
| B-F | acccAAGCTTcgTCCACCAAGTACTC | 131-281 |
| B-R | acggGGTACCCCACGTGGCGAGCAG |  |
| A1-F | acccAAGCTTcgATGGATCCACCCGAAATCAC | 1-80 |
| A1-R | acggGGTACCGTGGTCGCCCTTGGGGTG |  |
| A2-F | acccAAGCTTcgCCTCCCGCGCCGCACCCCAAG | 71-150 |
| A2-R | acggGGTACCGCGGAAGGCCATGTCGTTGTC |  |
| A3-F | acccAAGCTTcgCCTCCCGCGCCGCACCCCAAG | 71-115 |
| A3-R | acggGGTACCCGCGCGGCGCTGCATCTCCTTC |  |
| A4-F | acccAAGCTTcgACGTGTCCGAAGGAGATGC | 106-150 |
| A4-R | acggGGTACCGCGGAAGGCCATGTCGTTGTC |  |
| A5-F | acccAAGCTTcgCCTCCCGCGCCGCACCCCAAG | 71-90 |
| A5-R | acggGGTACCCACGTCTTTCCATTCCACCG |  |
| A6-F | acccAAGCTTcgGTGCTCAAGGCGGTGGAA | 81-105 |
| A6-R | acggGGTACCGGACTTGCACATGTCCGTGA |  |
| A7-F | acccAAGCTTcgCCGCATTTCTTCACGGACATG | 96-115 |
| A7-R | acggGGTACCCGCGCGGCGCTGCATCTCCTTC |  |
| A7-1F | agcttcgTTCTTCACGGACATGTGCAAGTCCACGTGTCCGAAGGAGATGCAGCGCggtac | 98-113 |
| A7-1R | cGCGCTGCATCTCCTTCGGACACGTGGACTTGCACATGTCCGTGAAGAAcga |  |
| A7-2F | agcttcgTTCACGGACATGTGCAAGTCCACGTGTCCGAAGGAGATGCAGCGCggtac | 99-113 |
| A7-2R | cGCGCTGCATCTCCTTCGGACACGTGGACTTGCACATGTCCGTGAAcga |  |
| A7-3F | agcttcgACGGACATGTGCAAGTCCACGTGTCCGAAGGAGATGCAGCGCggtac | 100-113 |
| A7-3R | cGCGCTGCATCTCCTTCGGACACGTGGACTTGCACATGTCCGTcga |  |
| A7-4F | agcttcgGACATGTGCAAGTCCACGTGTCCGAAGGAGATGCAGCGCggtac | 101-113 |
| A7-4R | cGCGCTGCATCTCCTTCGGACACGTGGACTTGCACATGTCcga |  |
| A7-5F | agcttcgATGTGCAAGTCCACGTGTCCGAAGGAGATGCAGCGCggtac | 102-113 |
| A7-5R | cGCGCTGCATCTCCTTCGGACACGTGGACTTGCACATcga |  |
| A7-6F | agcttcgTGCAAGTCCACGTGTCCGAAGGAGATGCAGCGCggtac | 103-113 |
| A7-6R | cGCGCTGCATCTCCTTCGGACACGTGGACTTGCAcga |  |
| A7-7F | agcttcgAAGTCCACGTGTCCGAAGGAGATGCAGCGCggtac | 104-113 |
| A7-7R | cGCGCTGCATCTCCTTCGGACACGTGGACTTcga |  |
| A7-8F | agcttcgTGCAAGTCCACGTGTCCGAAGGAGATGCAGggtac | 103-112 |
| A7-8R | cCTGCATCTCCTTCGGACACGTGGACTTGCAcga |  |
| A7-9F | agcttcgTGCAAGTCCACGTGTCCGAAGGAGATGggtac | 103-111 |
| A7-9R | cCATCTCCTTCGGACACGTGGACTTGCAcga |  |
| A7-10F | agcttcgTGCAAGTCCACGTGTCCGAAGGAGggtac | 103-110 |
| A7-10R | cCTCCTTCGGACACGTGGACTTGCAcga |  |
| A7-11F | agcttcgTGCAAGTCCACGTGTCCGAAGggtac | 103-109 |
| A7-11R | cCTTCGGACACGTGGACTTGCAcga |  |
| A7-12F | agcttcgTGCAAGTCCACGTGTCCGggtac | 103-108 |
| A7-12R | cCGGACACGTGGACTTGCAcga |  |
| A7-13F | agcttcgTGCAAGTCCACGTGTggtac | 103-107 |
| A7-13R | cACACGTGGACTTGCAcga |  |
| A7-14F | agcttcgTGCAAGTCCACGggtac | 103-106 |
| A7-14R | cCGTGGACTTGCAcga |  |

Note: The underlines indicate the restriction enzyme sites.

# The positions correspond to amino acid sites in F1L with accession number OQ686990.
